# Five-year effectiveness of short messaging service (SMS) for pre-diabetes

**DOI:** 10.1186/s13104-018-3810-y

**Published:** 2018-10-10

**Authors:** Carlos K. H. Wong, Shing-Chung Siu, Ka-Wai Wong, Esther Y. T. Yu, Cindy L. K. Lam

**Affiliations:** 1 Department of Family Medicine and Primary Care, The University of Hong Kong, Rm 1-01, 1/F, Jockey Club Building for Interdisciplinary Research, 5 Sassoon Road, Pokfulam, Hong Kong SAR, China; 2 Department of Medicine and Rehabilitation, Tung Wah Eastern Hospital, Hong Kong SAR, China

**Keywords:** Economic evaluation, Short messaging services, IGT, Pre-diabetes

## Abstract

**Objective:**

An observational post-randomized controlled trial (RCT) design was adopted to evaluate the long-term sustainability and maintenance of improved glycemic control, lipid profile, reduced progression to diabetes at 3-year following a 2-year short messaging service (SMS). We performed a naturalistic follow-up to the 104 participants of SMS intervention, a 2-year randomized controlled trial comparing the SMS to non-SMS for pre-diabetes. All participants were arranged screening for diabetes at 5-year assessment. Primary outcome of this post-RCT study was cumulative incidence of diabetes whereas secondary outcomes were the change in biometric data over a 5-year period.

**Results:**

After a mean 57-month follow-up, 19 (18.3%) were lost to follow-up after the RCT period. Progression to diabetes occurred in 20 and 16 patients among the intervention and control group respectively, with no significant between-group difference (8.06 and 7.31 cases per 100 person years, respectively; Hazard Ratio in the intervention group, 1.184; 95% confidence interval, 0.612 to 2.288; p-value = 0.616). No significant effect of SMS on reduction in diabetes was observed in overall and pre-defined subgroups. The SMS intervention preserved the clinical benefits within the trial period but failed to transform from treatment efficacy to long-term effectiveness beyond 2 years after intervention.

*Trail registration* ClinicalTrials.gov Identifier NCT01556880, retrospectively registered on March 16, 2012

## Introduction

Diabetes mellitus (DM) is a global epidemic issue with age-specific prevalence of 8.3% [1] and considered as undiagnosed in 45.8% of all DM cases [2], in which is likely to result in both cardiovascular and non-cardiovascular morbidity and mortality. Prediabetes is a precursor stage before DM, where abnormal glycose regulation including impaired glucose tolerance (IGT) and/or impaired fasting glucose (IFG) was observed. It was reported that approximately 5–10% of people with prediabetes convert to diabetic patients annually [3]. The identification of efficient and effective interventions for DM prevention is imperative at reducing the disease and economic burden attributable to DM and its complications. Thus, interventions are targeted to halt the progression of prediabetes to diabetes.

Different forms of treatment modalities are available for DM prevention among patients with pre-diabetes [4]. Effective interventions aiming at preventing DM include pharmacological interventions with oral antidiabetic drugs [5, 6], non-pharmacological lifestyle interventions with intensive training in diet and physical exercise [7], and more aggregative surgical interventions with bariatric surgery [8]. Despite American Diabetes Association (ADA) [9] and National Institute for Health and Care Excellence (NICE) [10] guidelines that advocated intensive lifestyle modification program for high-risk individuals with pre-diabetes, they have not been routinely performed in many clinical practice settings. The landmark multicenter randomized controlled studies (RCTs) such as Diabetes Prevention Program (DPP) [11] and Diabetes Prevention Program Outcomes Study (DPPOS) [12] in the US, Da Qing Diabetes Prevention Program in China [13, 14], Finnish Diabetes Prevention Study [15–17] demonstrated the long-term effectiveness of lifestyle modification intervention on the diabetes prevention among IGT patients. Collective evidence from systematic reviews [18, 19] illustrated that lifestyle interventions compared to placebo or control group were associated with significant reduction in relative risk of DM, despite the heterogeneity in lifestyle programs and duration of study and follow-up in those trials. More importantly, based on the long-term post-trial data from the DPP and DPPOS, participants in lifestyle intervention reduced significantly more diabetes incidence than those in metformin [12]. Therefore, non-pharmacological lifestyle modification intervention is recognized as a first-line treatment modality for DM prevention.

Lifestyle interventions in those with IGT were not only effective but also highly cost-effective in the long term [20, 21]. Evolution of technology overcomes challenges and barriers to deliver core contents of lifestyle modification through cellular phones and other electronic devices [9]. Ranging from reminder systems via short messaging service to tele-consultation, telemedicine strategies are helpful and useful, especially for patients who have difficulties in traveling to health care facilities due to long distances or disabilities [22]. The effectiveness of telemedicine on the management of diabetes has already been confirmed by two systematic reviews [22, 23], both of which found that telemedicine interventions significantly reduced haemoglobin A1c (HbA1c) of diabetic patients compared with usual care. Short messaging services (SMS) via cellular phones serves as a mode of knowledge delivery, and an effective mean to enhance lifestyle modification. Our within-trial report [24] indicated that the SMS intervention had beneficial effects on diabetes prevention at 12-month but protective effects were attenuated at 24-month. With regard to its cost-effectiveness, the SMS intervention was considered as cost-saving when compared to control group [25].

The objectives of this post-trial report were to observe glycemic control, blood pressure, waist circumstance, weight, and body mass index (BMI) levels after cessation of SMS trial, determine the long-term impact of SMS intervention on diabetes outcome, and evaluate the effectiveness of SMS for patients with pre-diabetes at 5-year.

## Main text

Study design and protocol have previously been described elsewhere [24]. In brief, 104 participants with pre-diabetes (i.e. IFG or/and IGT) who were accessible and received Chinese text messages by mobile phone were recruited from a project to screen for pre-diabetes and undiagnosed DM in Hong Kong. IFG was defined as a fasting plasma glucose level of 5.6–5.9 mmol/L. IGT was defined as a fasting plasma glucose level of < 7.0 mmol/L or 2-h post-load plasma glucose (2HPPG) of 7.8–11.0 mmol/L after a 75-g glucose load according to World Health Organization criteria [26]. Subjects were excluded if they had a history of DM, were on medicines known to alter glucose tolerance, were unable to read Chinese characters, and refused to take part in study.

All 104 participants were randomized either to 2-year SMS intervention or usual care without SMS reminder delivered by our research team, and were given booklets with information of pre-diabetes and diabetes by the research nurse. In the intervention group, text messages were sent three times a week, once per week and once per month within the first 3 months, the second 3 months, and the subsequent 18 months, respectively. At the trial end, diabetes onset in the SMS group was reduced by 38% when compared with control group [24]. After a mean 57-month follow-up (range 10–82 months), all participants were approached for consent to take part in this post trial study from September 2015 to April 2016. Electronic medical records were retrieved to obtain the diagnosis of event, anthropometric and blood measurements for those who had clinical reading and detailed events recorded within 1-year of assessment. For those recorded at the time beyond one-year of assessment, the research team arranged health examinations to obtain anthropometric and blood measurements.

Ethics approval for this post-trial study was obtained from the Institutional Review Board of the Hong Kong East Cluster of the Hospital Authority.

### Statistical analysis

Descriptive statistics were used to show the distribution of socio-demographic, occupational profile, lifestyle, clinical history, and to summarize the biometric data (weight, BMI, waist circumstance, blood pressure, lipid profile) of the SMS intervention and control patients. Significant differences between the two groups were compared by Chi square tests for categorical variables and independent t-tests for continuous variables.

Biometric data were analysed according to the intention-to-treat principle. Missing values at subjects who were lost to follow-ups (i.e. defaulted or withdrawal) were imputed with last observed value carried forward. Sensitivity analysis was performed on complete cases for biometric data. Repeated measures analysis of variance was conducted to assess differences in biometric data over the time and their interactions between groups.

Primary outcome of this observational study was the DM incidence. Kaplan–Meier estimates were used to calculate the cumulative proportion of patients who had a DM event (i.e. fasting glucose level ≥ 7.0 and/or 2HPPG ≥ 11.1 mmol/L). The hazard ratio (HR) of SMS intervention was estimated by Cox regression using overall 104 patients. Repeated analyses considering 14 pre-specified subgroups (based on age, gender, working shift based, regular exercise, family history of DM, history of high blood pressure, and BMI at baseline) were done to assess heterogeneity of treatment effects. The incidence rates of DM among overall sample and the pre-specified subgroups were reported.

All statistical analysis was performed using STATA Version 13.0 (StataCorp LP, College Station, Tex). All significance tests were two-tailed and findings with a p-value less than 0.05 were considered statistically significant.

### Results

**Fig. 1:**
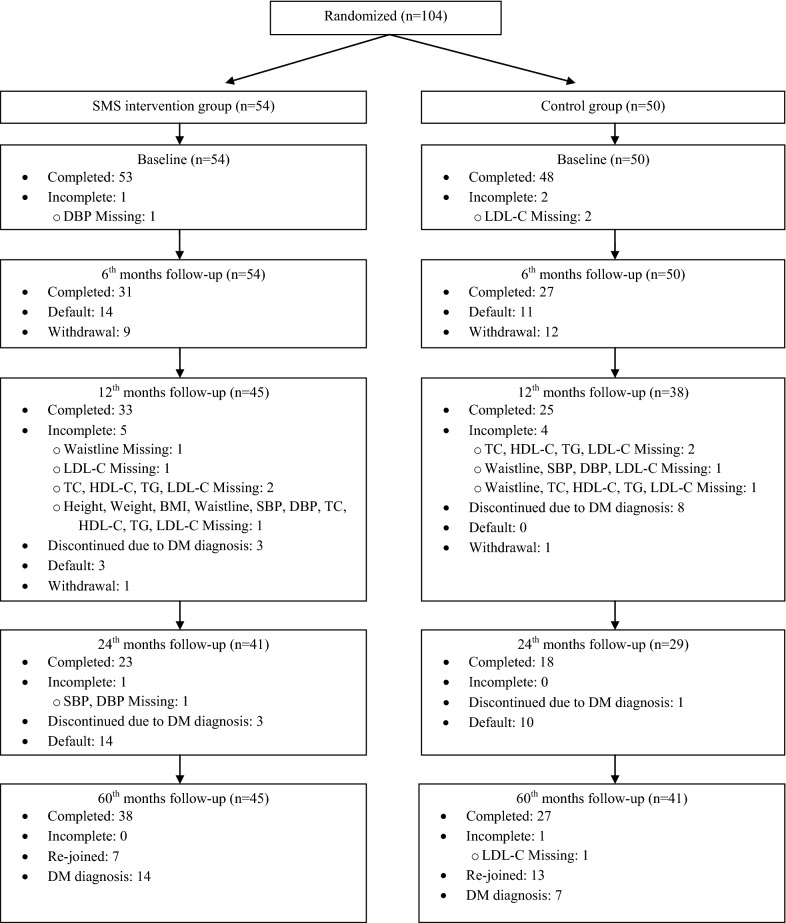
Flowchart on the subject allocation and participation in randomized controlled trial and post-trial follow-up

At trial randomization stage, 104 subjects were randomly assigned to either the SMS group or control group (Fig. 1). However, the majority of subjects in both groups were male (90.7% and 96.0%). At 60-month follow-up, 86 (65 subjects completed 24-month follow-up and 21 subjects whose were withdrawal in previous follow-ups) completed assessments whereas 21 subjects (14 in SMS and 7 in control group) had DM occurrence. The number of subjects progressing from pre-diabetes to DM was 36 (34.6%) over the 60 months.

Table 1 shows the effect of the SMS group on the change in the level of biometric data. No significant interactions between treatment group and time were found in all biometric factors. There were significant mean differences on weight, BMI, waist circumstance, total cholesterol (TC), high density lipoprotein-cholesterol (HDL-C) and low density lipoprotein-cholesterol (LDL-C) over time (p-value = 0.005; 0.007; 0.006; < 0.001; 0.014; < 0.001) in the intention-to-treat analysis. However, the mean changes in systolic blood pressure (SBP), diastolic blood pressure (DBP), Triglyceride (TG) and DM risk score were not significantly different between groups, over time. The interaction effect between groups and time on those changes were not significant.

**Table 1:** Effect of the SMS intervention on the change in the level of biometric data

	Time					Repeated measure ANOVA		
						p-value		
	Baseline	6 months	12 months	24 months	60 months	Group	Time	Group × time
Weight (kg)						0.109	0.005*	0.101
Control	72.32 ± 10.01	72.58 ± 10.29	72.30 ± 10.49	71.91 ± 10.88	71.33 ± 10.23			
Intervention	69.49 ± 10.52	69.01 ± 10.40	68.40 ± 10.19	68.47 ± 10.35	68.76 ± 10.88			
BMI (kg/m^2^)						0.121	0.007*	0.206
Control	26.25 ± 2.95	26.24 ± 2.99	26.28 ± 3.14	26.18 ± 3.27	25.97 ± 3.08			
Intervention	25.55 ± 2.94	25.31 ± 3.02	25.18 ± 3.10	25.11 ± 3.04	25.14 ± 3.33			
Waist (cm)						0.118	0.006*	0.998
Control	92.04 ± 8.05	91.78 ± 8.29	91.70 ± 8.33	91.72 ± 8.50	92.95 ± 7.85			
Intervention	89.86 ± 7.42	89.45 ± 7.22	89.38 ± 7.08	89.34 ± 7.47	90.82 ± 8.18			
SBP (mmHg)						0.582	0.599	0.796
Control	133.90 ± 16.45	135.18 ± 17.65	132.48 ± 19.13	133.74 ± 18.65	132.60 ± 17.36			
Intervention	136.54 ± 15.88	135.06 ± 17.16	135.46 ± 19.65	134.96 ± 16.31	134.05 ± 15.63			
DBP (mmHg)						0.999	0.140	0.148
Control	80.86 ± 11.04	80.34 ± 11.22	80.12 ± 13.02	79.74 ± 11.94	79.42 ± 12.00			
Intervention	80.32 ± 10.67	81.87 ± 17.94	77.76 ± 12.73	77.85 ± 11.64	81.74 ± 14.05			
TC (mmol/L)						0.259	<0.001*	0.946
Control	5.49 ± 0.93	NA	5.45 ± 0.99	5.42 ± 0.87	5.13 ± 0.85			
Intervention	5.35 ± 0.72	NA	5.24 ± 0.77	5.28 ± 0.90	4.96 ± 0.87			
HDL (mmol/L)						0.791	0.014*	0.389
Control	1.32 ± 0.39	NA	1.22 ± 0.27	1.21 ± 0.26	1.23 ± 0.31			
Intervention	1.28 ± 0.40	NA	1.24 ± 0.27	1.22 ± 0.25	1.30 ± 0.28			
TG (mmol/L)						0.242	0.222	0.540
Control	1.77 ± 1.09	NA	1.95 ± 1.90	1.93 ± 1.91	1.75 ± 1.22			
Intervention	1.71 ± 0.87	NA	1.61 ± 1.15	1.65 ± 1.20	1.41 ± 0.71			
LDL (mmol/L)						0.263	<0.001*	0.922
Control	3.47 ± 0.85	NA	3.50 ± 0.92	3.49 ± 0.80	3.13 ± 0.78			
Intervention	3.34 ± 0.70	NA	3.32 ± 0.72	3.33 ± 0.77	3.03 ± 0.82			

*ANOVA* analysis of variance, *BMI* body mass index, *SBP* systolic blood pressure, *DBP* diastolic blood pressure, *TC* total cholesterol, *HDL* high density lipoprotein, *TG* triglyceride, *LDL* low density lipoprotein, *NA* not applicable

Table 2 depicts the number and incidence rate of DM events and HR of SMS intervention. Progression to diabetes occurred in 20 and 16 patients among the intervention and control group respectively, with no significant between-group difference (8.06 and 7.31 cases per 100 person years, respectively; HR in the intervention group, 1.184; 95% confidence interval, 0.612 to 2.288; p-value = 0.616) (Table 2). In addition, there were no significant interactions among the pre-specified subgroups. The HRs in the subgroup of aged 65 or above and female were not applicable as the occurrence of DM event between intervention and control subjects in the subgroup of aged 65 or above were equal (1 vs. 1) while there was no occurrence of DM event in the control subjects (= 0) in the female subgroup.

**Table 2:** Number and incidence rate of DM events and hazard ratios of SMS intervention

	Intervention group (N = 54)				Control group (N = 50)				Hazard ratio	95% CI	p-value	p-value for interaction
Event of DM	Cumulative incidence		Incidence rate (cases/100 person-year)		Cumulative incidence		Incidence rate (cases/100 person-year)					
Sub-group	Cases with event	Rate (%)	Estimate	Person-year	Cases with event	Rate (%)	Estimate	Person-year				
Overall	20	37.04	8.06	248	16	32.00	7.31	219	1.184	(0.612, 2.288)	0.616	
Age, year												0.638
≥ 65	1	33.33	7.69	13	1	33.33	5.26	19	NA	NA	NA	
< 65	19	37.25	8.09	235	15	31.91	7.50	200	1.131	(0.574, 2.229)	0.723	
Gender												0.905
Male	17	34.69	7.46	228	16	33.33	7.73	207	1.035	(0.522, 2.052)	0.922	
Female	3	60.00	15.00	20	0	0.00	0.00	12	NA	NA	NA	
Working shift based												0.996
Yes	5	41.67	10.20	49	4	28.57	5.71	70	2.221	(0.564, 8.745)	0.254	
No	15	35.71	7.54	199	12	33.33	8.05	149	0.963	(0.451, 2.059)	0.923	
Regular exercise												0.692
Yes	4	26.67	5.88	68	5	25.00	5.49	91	1.102	(0.296, 4.112)	0.885	
No	16	41.03	8.84	181	11	36.67	8.59	128	1.114	(0.516, 2.407)	0.784	
Family history of DM												0.415
Yes	11	37.93	8.46	130	13	38.24	8.55	152	1.058	(0.473, 2.365)	0.891	
No	9	36.00	7.56	119	3	18.75	4.48	67	1.939	(0.522, 7.205)	0.323	
History of high blood pressure												0.378
Yes	4	50.00	11.76	34	2	18.18	5.00	40	2.287	(0.417, 12.54)	0.341	
No	16	34.78	7.48	214	14	35.90	7.82	179	1.037	(0.505, 2.131)	0.921	
BMI, kg/m^2^												0.257
≥ 25	12	42.86	9.16	131	11	36.67	9.24	119	1.022	(0.450, 2.318)	0.959	
< 25	8	30.77	6.84	117	5	25.00	5.00	100	1.476	(0.481, 4.531)	0.496	

*DM* diabetes mellitus, *BMI* body mass index, *CI* confidence interval, *NA* not applicable

### Discussions

This post-RCT report conferred the long-term effectiveness of a 2-year cellular phone-based SMS intervention in patients with pre-diabetes. One of the principal findings was that the immediate outcomes and diabetes outcome were highly comparable at the end of follow-up. Although the SMS intervention was effective in reducing DM events during the 24-month trial period, the reduction in DM events by SMS intervention was attenuated at 3 years after the cessation of trial. Small differences in cumulative DM incidence averted in SMS intervention failed to result in long-term benefits in DM prevention at 60-month of follow-up. The phenomenon of ‘legacy effect’ of SMS intervention on DM outcome was not observed in current intervention for pre-diabetes. Unlike the pragmatic RCTs like DPP [12], Da Qing Diabetes Prevention Program in China [13, 14], Finnish Diabetes Prevention Study [17], those trials demonstrated a legacy effect for lifestyle modification on prevention of DM for pre-diabetes occurred over a decade after intervention. Durability of protective effects lasted for SMS intervention group was one of the key determinants of relative effectiveness, in which was influenced by frequency of text messaging and duration of intervention. However, whether an intensification of intervention such as increased messaging frequencies or extended duration of intervention could achieve a long-term clinical benefit remained uncertain. Furthermore, advanced two-way interactive platform such as internet-driven social networks is alternative means to deliver lifestyle modification contents [9]. Whether those electronic platforms are effective and cost-effective vehicles to deliver lifestyle modification materials for DM prevention in comparison to SMS and traditional face-to-face approaches warranted further exploration.

Based on post-RCT data, the SMS intervention preserved the clinical benefits within the trial period but it failed to transform from treatment efficacy to long-term effectiveness beyond 2 years after intervention, and was not associated with significant reductions in diabetes prevention over 5 years. Possible reasons for the insignificant effectiveness of SMS compared to regular care in this study include sample size, representativeness of the sample and the durability of the SMS. Hence, further researches on whether increasing sample size or messaging frequencies, or extending duration of SMS for pre-diabetes could achieve a long-term clinical benefit are needed.

## Limitations

Although this analysis was conducted using randomized controlled trial data, there were several limitations requiring cautious interpretation of study findings. First, no routine yearly assessment was undertaken at post-trial period (from year 3 to 5), which may record a lagged onset date of DM from electronic medical records. For those diagnosed with DM, annual assessments would keep track of DM outcome on regular basis and thus preclude potential overestimation of the follow-up duration and the number of patients at risk of DM. Second, the missing data were handled by using last observed value. This method may not appropriate as it induced errors and thus affected the accuracy of long-term effectiveness. Third, this paper did not report the hypoglycaemic event, HbA1c and fasting plasma glucose values at baseline and follow-ups and, thus, failed to compare the effectiveness of two interventions on changes in glycaemia-related biometrics [22, 23]. In addition, the sample size of this study was relatively small and almost all of the participants were men, so that this study may not be able to give good and comprehensive estimates for the effectiveness analysis.

## Abbreviations

RCT: post-randomized controlled trial
SMS: short messaging service
HR: hazard ratio
DM: diabetes mellitus
IGT: impaired glucose tolerance
IFG: impaired fasting glucose
ADA: American Diabetes Association
NICE: National Institute for Health and Care Excellence
RCTs: randomized controlled studies
DPP: diabetes prevention program
DPPOS: Diabetes Prevention Program Outcomes Study
HbA1c: haemoglobin A1c
BMI: body mass index
2HPPG: 2-h post-load plasma glucose
ANOVA: analysis of variance
TC: total cholesterol
HDL-C: high density lipoprotein-cholesterol
LDL-C: low density lipoprotein-cholesterol
SBP: systolic blood pressure
DBP: diastolic blood pressure
TG: triglyceride
